# Captive Dwarf and Mouse Lemurs Have Variable Fur Growth

**DOI:** 10.3390/ani10081288

**Published:** 2020-07-28

**Authors:** Brooke Erin Crowley

**Affiliations:** 1 Department of Geology, University of Cincinnati, Cincinnati, OH 45221-0013, USA; brooke.crowley@uc.edu; Tel.: +1-513-551-7181; 2 Department of Anthropology, University of Cincinnati, Cincinnati, OH 45221-0380, USA

**Keywords:** Duke Lemur Center, *Cheirogaleus medius*, *Microcebus murinus*, seasonality, photoperiod, torpor

## Abstract

**Simple Summary:**

Next to nothing is known about fur or hair growth for any primate species aside from humans. Researchers have typically assumed that fur and hair growth is constant, but the available data suggest this is not the case. I investigated how quickly fur grows for two closely related species of small-bodied lemur, dwarf lemurs, and mouse lemurs at the Duke Lemur Center. I found that fur growth varied considerably both within and among individuals. Additionally, growth was overall slower and quite seasonally variable for dwarf lemurs. Seasonal fluctuations in fur regrowth likely reflect changes in metabolism related to photoperiod, a phenomenon that is widespread among vertebrates. Variable fur growth is problematic for any study that uses keratin to investigate seasonal changes in diet or health. Further research examining how variable fur and hair growth is across a larger array of species, between sexes, and across seasons is needed.

**Abstract:**

Researchers typically assume constant fur and hair growth for primates, but the few studies that have investigated growth explicitly suggest this may not be the case. Instead, growth may vary considerably among individuals and across seasons. One might expect this variability to be most pronounced for species that have seasonally variable activity patterns (e.g., Madagascar’s Cheiorogaleidae). In particular, dwarf lemurs (*Cheirogaleus* spp.) undergo considerable changes in their daily activity levels (torpor) in the austral fall, when nights get shorter. I monitored regrowth of shaved fur patches for eight adult captive fat-tailed dwarf lemurs (*Cheirogaleus medius*) and gray mouse lemurs (*Microcebus* *murinus*) on a bi-weekly basis for 21 months in total. Regrowth varied considerably both within and among individuals. Overall, fur regrew in spurts and was faster for mouse lemurs (0–14 to 215–229 days) than dwarf lemurs (27–40 to 313–327 days). There were significant differences between species and an obvious influence of season for dwarf lemurs, but no clear influence of shave location, age, or sex. Similar trends have been previously reported for captive lemurids, suggesting that seasonal fur growth may be widespread across Lemuroidea. Researchers are cautioned against using primate fur or hair to investigate variables confounded by seasonality (such as diet and body condition) until patterns of growth are better understood.

## 1. Introduction

Researchers are increasingly incorporating analytical methods for monitoring behavior, body condition, and health that can complement observational data. Fur and hair keratin record a wealth of information about an individual’s recent history, and both isotopic and hormonal analyses have emerged as minimally-invasive tools for identifying competition and niche partitioning, interpreting environmental conditions, and monitoring both short-term and chronic stress, reviewed in [1,2]. However, these approaches require knowledge of the amount of time represented in a particular fur or hair strand, which in turn requires a solid understanding of fur and hair growth. Without knowing the rate of growth or, on a basic level, whether growth is continuous or seasonal, one cannot confidently estimate the temporal window preserved in a given sample, which in turn limits the utility of fur or hair for tracking dietary intake, nutrition, and hormone levels among seasons and years, e.g., [3,4].

There has been more than a century of research on fur and hair growth across Mammalia, e.g., [5,6,7,8,9,10,11], reviewed in [12]. Yet, the majority of this has work been focused on animals that grow and shed seasonal coats, and on hair loss. Hair or fur growth for animals with more continuously growing hair, such as primates, has been much more limited. There has been some work on humans, e.g., [12,13,14,15,16,17], but remarkably little attention has been paid to fur and hair growth in non-human primates. The few exceptions are a handful of studies by Uno and colleagues on stump-tailed macaques (*Macaca speciosa*) that were focused on alopecia [18,19,20], a study by Fourie and colleagues [3] that demonstrated considerable variability in fur and hair growth for a handful of captive individuals (N = 1–3) from a range of old-world monkey and ape species (Allen’s swamp monkey, *Allenopithecus nigroviridis*; golden-bellied mangabey, *Cercocebus chrysogaster*; gelada baboon, *Theropithecus gelada*; western lowland gorilla, *Gorilla gorilla*; yellow-cheeked crested gibbon, *Hylobates gabriellae*; and Siamang, *Symphalangus syndactylus*), and a study by Pereira and colleagues [21] that demonstrated seasonal fur growth for captive ring-tailed lemurs (*Lemur catta*) and red-fronted brown lemurs (*Eulemur fulvus rufus*).

I examined fur growth for two cheirogaleid lemur species, fat-tailed dwarf lemurs (*Cheirogaleus medius*) and gray mouse lemurs (*Microcebus murinus*), housed at the Duke Lemur Center (DLC) in Durham, North Carolina, USA. Hair and fur both pertain to the same physical structure, but the terms are typically used to refer to follicles that produce longer strands (hair) or shorter body cover (fur). Authors have used both terms for lemurs. Given that cheirogaleids have short body cover, I used fur in this study. I chose these small-bodied lemurs for two reasons. First, cheirogaleids exhibit the most pronounced seasonal metabolic changes of any primate [22], and thus are well-suited for examining potential seasonal fluctuations in fur growth. Second, both genera (but particularly mouse lemurs) have been the study subjects of both isotopic and hormonal research over the past decade, e.g., [23,24,25,26,27,28]. Researchers have typically assumed constant fur growth, and estimated growth rates based on ad-hoc observations in the field, e.g., [4], or data for murid rodents, e.g., [26,28]. However, as noted by Rakotoniaina and colleagues [4]: “At present, the lack of precise information on hair growth rate in *M. murinus* limits our estimation about the period of accumulation of cortisol recorded with the hair sample”. Given the growing interest in investigating seasonal trends in diet, e.g., [23,26,27], monitoring seasonal variability in stress hormones, e.g., [4], and using cheirogaleids as proxies for tracking baseline isotopic differences among habitats in Madagascar, e.g., [24,25], establishing how their fur grows is both necessary and timely.

On the basis of observed fur growth for lemurids and most other mammals (described in more detail below), I anticipated that fur growth for captive cheirogaleids at the DLC would vary among seasons, with faster regrowth occurring when the lemurs are most active (i.e., between North Carolina’s summer solstice and fall equinox), and slow to negligible regrowth during winter months, when lemurs undergo brief to extended bouts of suppressed metabolic rate (i.e., torpor). I further expected that dwarf lemurs, which have larger bodies and undergo more pronounced torpor, would exhibit slower fur growth than mouse lemurs.

### Factors Affecting Fur and Hair Growth

Fur and hair growth is primarily regulated by hormones, but may also be affected by nutrition, and possibly shaving. Sex hormones (particularly androgens) and thyroid hormones (e.g., thyroxine) are the most important players [10,12,29,30]. Seasonal fluctuations in growth are most pronounced in mammals with seasonal coats, but have also been observed in humans [16,31,32,33,34]. These are triggered by changes in melatonin secretion, which in turn is driven by photoperiod, and to a lesser extent temperature [5,6,7,8,9,10,11,12]. An adequate intake of both carbohydrates (particularly glycogen) and proteins (particularly the amino acids cysteine and methionine) is very important for functioning follicles and keratin synthesis, as reviewed in [35]. There are also a host of vitamins and minerals that influence growth, but mostly these affect how a particular strand grows (e.g., wavy or straight; pigmentation) or stimulate hair loss, reviewed in [35]. There is mixed evidence about the degree to which shaving affects growth. Simply shaving skin (as opposed to plucking or chemically removing hair) should not affect the growth cycle, reviewed in [36] and there is no measurable effect of repeated shaving on growth of leg or chest hair in adult men [16,17]. However, growth increased following shaving both in mice [37] and in human beards [13,14]. Both of these studies found that initial regrowth was rapid, but then slowed.

## 2. Materials and Methods

### 2.1. Sample Collection

I monitored fur growth and body mass for four fat-tailed dwarf lemurs and four gray mouse lemurs housed at the DLC between February 2017 and October 2018. Two males and two females were included from each species. Details about each individual are provided in Table 1. None of the study subjects were reproductively active and all were considered healthy adults (mouse lemurs are reproductively mature at the DLC by six months). However, one female mouse lemur, Nettle (MF1), suffered a self-inflicted injury and had to be euthanized part way through the study.

**Figure 1:**
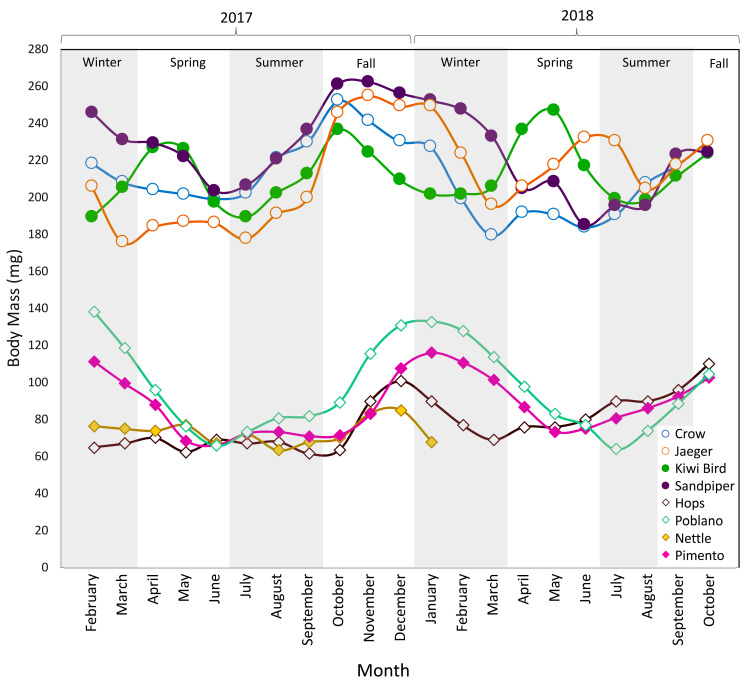
Monthly body mass measurements for each individual. Dwarf lemurs are circles and mouse lemurs are diamonds. Males are open symbols; females are filled. If more than one measurement was taken within the same month, the weights were averaged.

Body mass is recorded regularly for all DLC lemurs (Figure 1; Table S1). There is no standard method for collecting primate fur or monitoring regrowth. Study subjects were briefly (<3 min) removed from their sleep sites during their “day phase” (i.e., white lights on) and manually restrained by trained DLC staff. A nasal hair trimmer was used to shave a small patch of fur (ca. ½″ diameter; 12–40 mg) from each lemur’s hip, thigh, tail base, or side of torso on a bi-monthly basis starting in February 2017 and ending in March 2018 (eight shaves total, see Table 2). I focused on the base of the tail region because it is where I have previously sampled fur for isotopic research [25,26,27], and is not typically chewed or groomed. Following each shave, regrowth was measured on a bi-weekly basis using calipers (study subjects were handled as described above). Following Fourie et al. [3], fur was considered completely regrown when a shaved patch was no longer discernable from the surrounding fur. There were some cases where regrowth was patchy, or fur mostly regrew to the length of original, surrounding fur but the borders of the patch could still be discerned for several weeks. I erred on the side of slightly underestimating regrowth rates and used the first date after which fur length did not change for summary data and statistical analyses.

DLC cheirogaleids vary their activity levels across the year. During North Carolina’s austral fall and winter, both mouse and dwarf lemurs undergo torpor, which is a period of reduced activity closely tied to body mass and fat storage. During torpor bouts, body temperature, heart rate, and brain activity reduce dramatically. Torpor can be visually identified by reduced respiratory rate, typical posture (curled up in a ball, oftentimes outside of the nest), or sluggish movement and/or shivering [22]. Individual activity levels are not monitored closely at the DLC. I therefore only have limited observations of torpor-like behavior for three of the four dwarf lemurs included in this study (Table S2). No observation data are available for Crow (CM1) or the mouse lemurs.

### 2.2. Housing Conditions

DLC Cheirogaleids are fed commercially available primate chow, fruits, vegetables, and mealworms. For the majority of the study period, study subjects were housed in the DLC nocturnal building in enclosures ranging in size from 0.68 to 1.57 m^3^ either singly, or in social groups of up to three individuals. Temperature was set at 78 °F (25.6 °C) year-round, but outside ambient temperatures impacted this somewhat. The general sensor for the building showed daily temperatures ranged from 72 to 82 °F (night-time to heat of day), with most temperatures concentrating around 77–79 °F. The photoperiod for nocturnal lemurs at the DLC follows that of North Carolina but is offset to allow researchers to observe subjects while active (see Table S3). From April to June 2017, all cheirogaleids were moved to an alternate housing area while their normal space was renovated. Individuals were exposed to comparable temperature and light but lived in more crowded conditions and therefore may have experienced higher stress levels. In order to assess the degree to which increased stress might have affected fur growth rates, the study included the same window of time in 2018, with lemurs living in normal housing conditions.

### 2.3. Statistical Analysis

I used non-parametric Kruskal–Wallis analyses to check for the effects of species, individual, age, and shave number on fur regrowth time. I only worked with total time for regrowth (a single value) for each shave for each individual. Total N was 8 samples for most individuals, but several data points were missing due to Nettle’s death and the exclusion of two unusual samples for Poblano (described in more detail below). These analyses were followed up with Steel-Dwass all pairs post-hoc analyses when appropriate. I further disentangled the possible influence of sex, species, and time of shave (season or month) on fur growth using linear mixed models (lmm) that account for individual as a random effect. I also used lmm to test for the influence of shave location (“hip/thigh”, “side”, and “base of tail”), and the combined influence of shave location and species (again accounting for individual as a random effect). Including more variables in these models was not possible due to lost degrees of freedom. Given the small sample size, I recognize that interpreting results should be done with caution. Nevertheless, they are useful for confirming visual assessments of trends in the data. I defined seasons based on the typical solar cycle for North Carolina: “winter” is between the winter solstice and the spring equinox; “spring” is between the Spring equinox and the summer solstice; “summer” is between the summer solstice and the fall equinox; and “fall” is between the fall equinox and the winter solstice. All analyses were conducted in JMP Pro 14.0 with significance set at α = 0.05.

## 3. Results

Despite ample food availability and reasonably constant temperatures, there were pronounced fluctuations in body mass for both species for the duration of the study (Figure 1). Overall, masses were lowest in late spring to early summer and highest in fall–winter, but there were some clear deviations from these trends. First, in both years of the study, Kiwi Bird (CF1) retained a relatively low body mass in winter and then experienced a brief increase in mass in spring. Second, Nettle (MF1) and Hops (MM1) had low body masses in winter during the first year of the study, and relatively small increases in mass during the second fall/winter.

**Figure 2:**
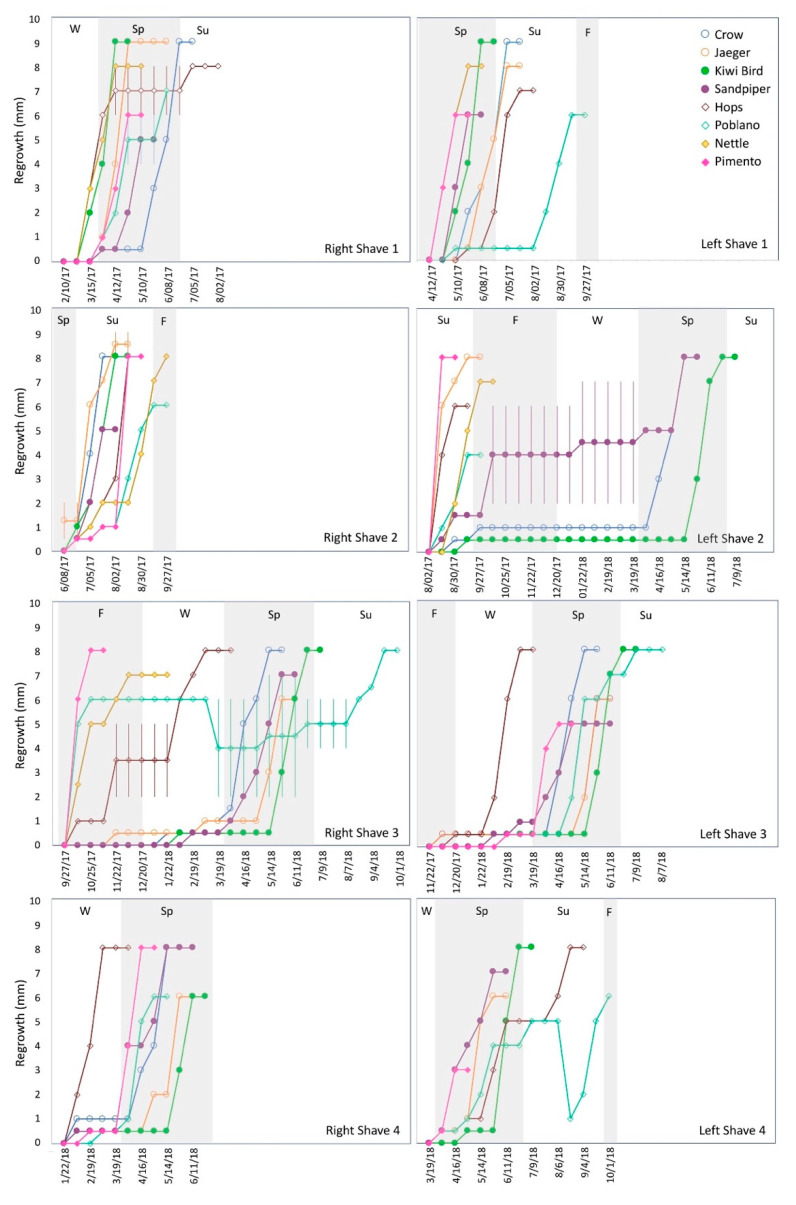
Fur regrowth for each shave and individual. Dwarf lemurs are circles and mouse lemurs are diamonds. Males are open symbols; females are filled. Error bars represent the range of length measurements for patchy regrowth (Supplementary Table S4). Shaded areas delineate different seasons (W = Winter; Sp = Spring; Su = Summer; F = Fall). No data are available for Nettle’s LS3, RS4, or LS4.

**Figure 3:**
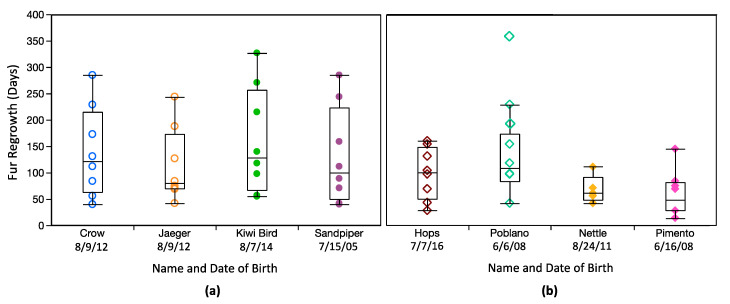
Box plots showing fur regrowth times for each (**a**) dwarf lemur, and (**b**) mouse lemur. There are no differences in regrowth rate among individuals in either species (*p* > 0.05). Boxes represent 25 and 75% quartiles, and whiskers contain 1.5 times the interquartile range. Measurements for Poblano’s RS3 (hip/thigh) and LS4 (base of tail) are shown but excluded from box plots and statistical comparisons. Symbology corresponds with Figure 1 and Figure 2. Males are open symbols; females are closed symbols.

Table 2 summarizes time for complete regrowth for each shave for each individual, while bi-weekly measurements for each shave for each individual are provided in Figure 2 and Supplementary Table S4. Fur regrowth varied considerably both within and among individuals. In most cases, regrowth was patchy or occurred non-linearly (slow initially followed by rapid regrowth; Figure 2; Supplementary Table S4). Poblano (MM2) had overall slower regrowth than other mouse lemurs (Table 2; Figure 3). Negative measurements for both RS3 and LS4 suggest he chewed on his regrowing fur (Figure 2; Table S4), and I excluded these two shaves from statistical analyses. Regrowth was also quite slow for Poblano’s LS3. However, I included this shave in statistical analyses as there was nothing to indicate it was compromised (e.g., no negative growth; Figure 2). Data are missing for Nettle’s LS3, RS3, and RS4 due to her necessary euthanasia in January 2018. Excluding RS3 and LS4 for Poblano, time for regrowth ranged from 0–14 days to 215–229 days for mouse lemurs, and 27–40 days to 313–327 days for dwarf lemurs (Table 2 and Table 3).

**Figure 4:**
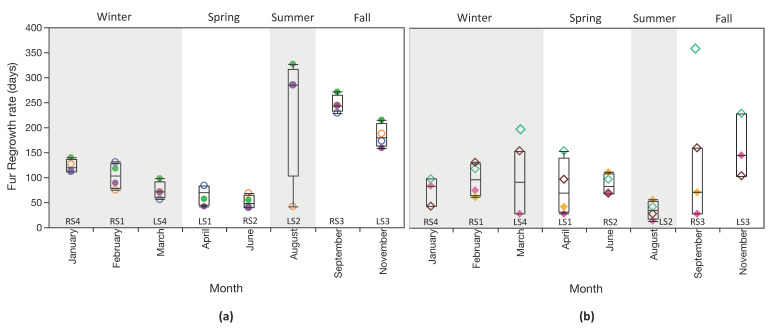
Box plots comparing regrowth among shaves done in different months for: (**a**) dwarf lemurs and (**b**) mouse lemurs. Boxes represent 25 and 75% quartiles, and whiskers contain 1.5 times the interquartile range. Measurements for Poblano’s RS3 (September 2017) and LS4 (March 2018) are shown but excluded from box plots and statistical comparisons. Symbology corresponds with Figure 1 and Figure 2. Males are open symbols; females are closed symbols.

There were significant differences in regrowth between species (χ^2^ = 4.7, df = 1, *p* = 0.030), with overall more rapid regrowth for mouse lemurs (Table 3; Figure 3). I found no influence of age on fur regrowth (χ^2^ = 10.64, df = 6; *p* = 0.10), and no statistically significant differences among individuals within either species (*p* > 0.05 for both taxa) despite highly variable regrowth, particularly for dwarf lemurs. These results may reflect my limited sample size and should be viewed with caution. There was a very clear seasonal influence on regrowth, at least for dwarf lemurs (Figure 4), with significant differences in regrowth time for shaves conducted in different months (χ^2^ = 22.3, df = 7, *p* = 0.0023; Table 3). Post-hoc analyses failed to identify any pairwise differences, but there are still quite distinct apparent trends. spring (LS1 and RS2) regrew rapidly while fall shaves (RS3 and LS3) regrew much more slowly (Figure 2 and Figure 4; Table 3). Regrowth was highly variable for LS2 (conducted on 2 August 2017). Mouse lemurs also exhibited variable fur regrowth, but shaves were statistically indistinguishable (χ^2^ = 9.7, df = 7, *p* = 0.21), and seasonal variability was less obvious (Figure 2 and Figure 4; Table 3). There was no noticeable influence of housing conditions on fur growth. Regrowth time March 2018; normal housing conditions) were indistinguishable (Figure 2 and Figure 4; Table 2 and Table 3).

A visual assessment of fur regrowth suggests no clear influence of sex (Figure 3). A linear mixed model (lmm) that included sex, species, and season as fixed variables (and individual as a random variable), confirmed that season and species are the primary factors influencing fur regrowth (Supplementary Table S5). Sex alone was not a significant variable.

Season, and the interaction between species and season, were the most important fixed variables, followed by species, the interaction between sex and season, and the interaction between species and sex. Including month rather than season resulted in a slightly weaker lmm (higher corrected Akaike information criterion), and only month, and the interaction between species and month, were significant (Supplementary Table S5). Given the small sample size, these results should be viewed cautiously, but they suggest that sex is not a significant predictor of fur growth in cheirogaleids.

**Figure 5:**
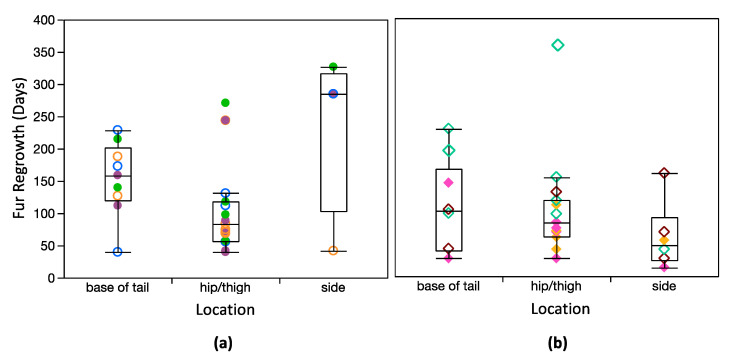
Box plots comparing regrowth among simplified shave locations for: (**a**) dwarf lemurs and (**b**) mouse lemurs. Boxes represent 25 and 75% quartiles, and whiskers contain 1.5 times the interquartile range. Measurements for Poblano’s RS3 (hip/thigh) and LS4 (base of tail) are shown but excluded from box plots and statistical comparisons. Symbology corresponds with Figure 1 and Figure 2. Males are open symbols; females are closed symbols.

Lastly, I verified that length of regrown fur was similar among shave locations for both species (Table 2), and there were no clear differences in regrowth rate among shave locations (Figure 5). A mixed model that included just shave location as the fixed variable (and individual as a random variable) suggests that shave location has no influence on fur growth (Supplementary Table S6). When species, and the interaction between species and shave location, were also included, the model was slightly stronger (lower corrected Akaike information criterion), but only species, and the interaction between species and shave location, were significant explanatory variables.

## 4. Discussion

Fur regrowth was remarkably variable for cheirogaleid lemurs at the DLC. Overall, dwarf lemurs exhibited slower growth (Figure 3; Table 3), but there was also a strong interaction between species and season (Supplementary Table S5). There was no obvious influence of shaving on fur growth. Regrowth was highly variable, but in many cases, initial regrowth of a shaved patch was slow (Figure 2). These results are largely consistent with those previously reported for captive lemurids, and also align with previous research that found shaving has no effect on the growth of leg or chest hair in adult men [16,17]. However, they contrast with studies that have reported rapid initial regrowth of shaved areas for mice and human faces [13,14,37].

Regrowth was also comparable for shaves done in different locations. Humans certainly have variable hair growth in different regions of the body (e.g., scalp, facial, and body hair), and perhaps if more diverse body regions were shaved (e.g., head and belly), I would have observed more variable regrowth rates among shave locations for cheirogaleids. To the best of my knowledge, comparative information is not available for other non-human primates. However, fur growth also differs among the flank, shoulder, and forehead of dogs [38]. Hale and Ebling [39] found that both fur length and growth rate differed among three body regions (ventral, dorsal, and mid-flank) for female rats, but these differences were not drastic. Total fur length varied from 10 to 20 mm (with shortest patches on the belly and longest patches on the back) and regrowth rates varied from 14 to 21 days. While growth was slightly quicker for shorter fur, in reality, longer dorsal hairs had to grow more rapidly in order to have a similar overall regrowth time.

Other variables, including age, sex, and genetic relatedness appeared to have a negligible, if any, influence on cheirogaleid fur growth. All four dwarf lemurs had quite variable fur regrowth despite differences in age, sex, and their genetic relatedness (Figure 3; Table 3). Crow and Jaeger are fraternal twins, yet their fur growth patterns were not particularly comparable (Figure 2; Supplementary Table S4). Similarly, although Kiwi Bird is Sandpiper’s daughter, these two lemurs also did not show especially comparable fur growth trends. Overall, these results are in agreement with Hamilton [15], who demonstrated that while genetic relatedness may play a role in growth rates for human facial and axillary hair, it is only appreciable for identical twins.

Dwarf lemurs, in particular, had seasonally variable fur regrowth, with the slowest growth for shaves done in late summer and fall (Figure 2 and Figure 4; Table 3). Seasonal fluctuations in fur regrowth likely reflect changes in metabolism related to photoperiod, a phenomenon which is widespread among birds, mammals, and even some ectothermic lizards [9]. Dwarf lemurs are the only primates that are obligate hibernators [40,41]. Mouse lemurs are more variable in their activity levels; they remain active in habitats with warm winters but can undergo prolonged hibernation in highly seasonal habitats, reviewed in [42,43]. Hibernation is expressed by torpor bouts that can last more than a week at a time in wild cheirogaleids, but shorter bouts are more frequent in captive lemurs. DLC dwarf lemurs tend to exhibit torpor-like behavior between November and February, and some individuals start slowing down in mid-October (DLC staff personal observation). This was true for both Jaeger (CM2) and Kiwi Bird (CF1) during the study (Table S2). Yet, somewhat surprisingly, slower fur growth was observed even earlier than this. In particular, highly variable regrowth was observed for LS2 (2 August 2017; Figure 2; Table 2 and Table 3). It would thus appear that dwarf lemurs may experience reduced metabolism for longer periods than is obvious from their sleeping behavior (i.e., extending beyond the spring and fall equinoxes) or body mass records.

Overall, fur growth for cheirogaleids, and dwarf lemurs in particular, is remarkably similar to reported trends for larger-bodied lemurids at the DLC [21]. Both male and female ring-tailed lemurs and red-fronted brown lemurs had rapid regrowth occurring between the summer solstice and fall equinox and negligible growth between the winter solstice and spring equinox regardless of whether they were living indoors or in a forest enclosure. The authors of the study mentioned that ruffed lemurs (*Varecia* spp.) at the DLC showed similar trends but did not show the data. Seasonal changes in fur growth were coincident with food intake, subcutaneous fat deposition, body mass, and hormone levels (insulin-like growth factor one and thyroxine). While sample sizes are small, and generated exclusively from captive individuals, they suggest that seasonal fluctuations in fur growth may be a widespread phenomenon across the Lemuroidea. This makes sense when one considers where lemurs evolved. Seasonality is pronounced across Madagascar. All lemur species have low metabolisms compared to other mammals, and exhibit changes in their metabolism and core temperatures to cope with seasonal shifts in temperature, as well as food and water availability [44]. Rapid fur growth for nocturnal lemurs when days are long and nights are short makes sense when one considers that longer days in Madagascar are also typically tied to warmer temperatures and increased precipitation, and that this in turn is linked with increased availability of fruits and arthropods, which make up the bulk of cheirogaleid diet, reviewed in [42,45]. Day length is also tied to seasonality in reproduction (and increased hormone production) in more seasonal habitats on the island, e.g., [43]. It would not be surprising if fluctuations in sex hormone production has some influence on fur growth for cheirogaleids given that they play a central role in human hair growth, reviewed in [10,12,29].

Variable fur growth for lemuroids aligns with the rather patchy data available for other non-human primates [3], as well as humans, e.g., [12,13,14,15,16,17]. The take home message here is that fur and hair growth can vary considerably among individuals, and growth may be quite seasonal even if a species does not grow and shed seasonal coats. Seasonally variable growth has important ramifications for any researcher interested in using fur or hair to assess diet, nutrition, and short-term stress. Samples collected in different seasons may record different windows of time. Additional work examining hair and fur growth in more detail across a larger array of species, and establishing how closely known changes in diet and body condition are reflected in keratin isotopes and hormone levels during different seasons is needed for both captive and wild populations.

## 5. Conclusions

In summary, fur growth for cheirogaleid lemurs is variable, and the accruing data suggest that variable fur and hair growth may be the norm for other primates, including humans. Fur and hair growth in non-human primates is a subject that has been largely ignored and there are no data on fur or hair growth for the vast majority of primate species. Researchers should keep this knowledge gap in mind when conducting isotopic, hormonal, and nutritional research as fur and hair may be seasonally biased in the information they preserve.

## Figures and Tables

**Table 1:** Individuals included in this study. Date of birth is presented as month/day/year.

Species	Sex	Name	Date of Birth	Notes
*Cheirogaleus medius*	Male	Crow	8/9/12	Crow and Jaeger are twins
	Male	Jaeger	8/9/12	Crow and Jaeger are twins
	Female	Sandpiper	7/15/05	
	Female	Kiwi Bird	8/7/14	Kiwi is Sandpiper’s daughter
*Microcebus murinus*	Male	Poblano	6/6/08	
	Male	Hops	7/7/16	
	Female	Pimento	6/16/08	
	Female	Nettle	8/24/11	Nettle incurred a mortal self-inflicted injury in January 2018

**Table 2:** Regrowth time for each shave for each individual. Dates are presented as Month/Day/Year.

Taxon	Name	Sex	Shave ID ^1^	Location	Date	Month	Season ^2^	Time to Full Regrowth (days)	Length (mm)	Regrowth Rate (mean ± 1σ; mm/day) ^5^
*Cheirogaleus*	Crow	Male	RS1	Hip/thigh	02/10/2017	February	Winter	<131	9	0.07 ± 0.11
			LS1	Hip/thigh	04/12/2017	April	Spring	<84	9	0.09 ± 0.11
			RS2	Base of tail	06/08/2017	June	Spring	<40	8	0.15 ± 0.11
			LS2	Side	08/02/2017	August	Summer	<285	8	0.03 ± 0.06
			RS3	Base of tail	09/27/2017	September	Fall	<229	8	0.03 ± 0.07
			LS3	Base of tail	11/22/2017	November	Fall	<173	8	0.04 ± 0.08
			RS4	Close to base of tail	01/22/2018	January	Winter	<112	8	0.06 ± 0.10
			LS4	Hip, superior to LS2	03/19/18	March	Winter	<56	8	0.11 ± 0.11
	Jaeger	Male	RS1	Hip/thigh	02/10/2017	February	Winter	<75	9	0.08 ± 0.13
			LS1	Hip/thigh	04/12/2017	April	Spring	<84	8	0.08 ± 0.09
			RS2	Hip/thigh	06/08/2017	June	Spring	<69	8.5	0.12 ± 0.13
			LS2	Side	08/02/2017	August	Summer	<42	8	0.14 ± 0.19
			RS3	Hip/thigh	09/27/2017	September	Fall	<244	6	0.02 ± 0.06
			LS3	Base of tail	11/22/2017	November	Fall	<188	6	0.03 ± 0.07
			RS4	Close to base of tail	01/22/2018	January	Winter	<127	6	0.04 ± 0.09
			LS4	Hip, superior to LS2	03/19/2018	March	Winter	<71	6	0.07 ± 0.11
	Sandpiper	Female	RS1	Hip/thigh	02/10/2017	February	Winter	<89	8	0.05 ± 0.05
			LS1	Hip/thigh	04/12/2017	April	Spring	<42	9	0.11 ± 0.12
			RS2	Hip/thigh	06/08/2017	June	Spring	<40	8	0.09 ± 0.10
			LS2	Side	08/02/2017	August	Summer	<285	8	0.03 ± 0.06
			RS3	Hip/thigh	09/27/2017	September	Fall	<244	8	0.03 ± 0.05
			LS3	Close to base of tail	11/22/2017	November	Fall	<159	8	0.03 ± 0.04
			RS4	Close to base of tail	01/22/2018	January	Winter	<112	6	0.06 ± 0.10
			LS4	Hip, superior to LS3	03/19/2018	March	Winter	<71	8	0.08 ± 0.06
	Kiwi Bird	Female	RS1	Hip/thigh	02/10/2017	February	Winter	<118	5	0.07 ± 0.09
			LS1	Hip/thigh	04/12/2017	April	Spring	<57	6	0.12 ± 0.14
			RS2	Hip/thigh	06/08/2017	June	Spring	<55	5	0.12 ± 0.10
			LS2	Side	08/02/2017	August	Summer	<327	8	0.02 ± 0.07
			RS3	Hip/thigh	09/27/2017	September	Fall	<271	7	0.03 ± 0.07
			LS3	Base of tail	11/22/2017	November	Fall	<215	5	0.04 ± 0.08
			RS4	Close to base of tail	01/22/2018	January	Winter	<140	8	0.04 ± 0.08
			LS4	Hip, superior to LS3	03/19/2018	March	Winter	<98	7	0.07 ± 0.13
*Microcebus*	Hops	Male	RS1	Hip/thigh	02/10/2017	February	Winter	<131	8	0.05 ± 0.08
			LS1	Hip/thigh	04/12/2017	April	Spring	<97	7	0.06 ± 0.10
			RS2	Side	06/08/2017	June	Spring	<69	8	0.10 ± 0.13
			LS2	Side	08/02/2017	August	Summer	<28	6	0.14 ± 0.14
			RS3	Side	09/27/2017	September	Fall	<160	8	0.04 ± 0.07
			LS3	Base of tail	11/22/2017	November	Fall	<104	8	0.07 ± 0.10
			RS4	Close to base of tail	01/22/2018	January	Winter	<43	8	0.11 ± 0.11
			LS4	Hip, same location as LS3	03/19/2018	March	Winter	<154	8	0.05 ± 0.06
	Poblano	Male	RS1	Hip/thigh	02/10/2017	February	Winter	<118	7	0.06 ± 0.08
			LS1	Hip/thigh	04/12/2017	April	Spring	<154	6	0.03 ± 0.06
			RS2	Hip/thigh	06/08/2017	June	Spring	<97	6	0.05 ± 0.06
			LS2	Side	08/02/2017	August	Summer	<42	4	0.07 ± 0.06
			RS3	Hip/thigh	09/27/2017	September	Fall	<356 ^3^	8	0.02 ± 0.08
			LS3	Base of tail	11/22/2017	November	Fall	<229	8	0.03 ± 0.07
			RS4	Close to base of tail	01/22/2018	January	Winter	<98	6	0.05 ± 0.10
			LS4	Base of tail	03/19/2018	March	Winter	<196 ^3^	6	0.03 ± 0.11
	Pimento	Female	RS1	Hip/thigh	02/10/2017	February	Winter	<75	6	0.07 ± 0.09
			LS1	Hip/thigh	04/12/2017	April	Spring	<28	6	0.14 ± 0.12
			RS2	Hip/thigh	06/08/2017	June	Spring	<69	8	0.10 ± 0.20
			LS2	Side	08/02/2017	August	Summer	<14	8	0.29 ± 0.40
			RS3	Hip/thigh	09/27/2017	September	Fall	<28	8	0.19 ± 0.22
			LS3	Base of tail	11/22/2017	November	Fall	<145	5	0.03 ± 0.08
			RS4	Hip/thigh	01/22/2018	January	Winter	<84	8	0.08 ± 0.13
			LS4	Base of tail	03/19/2018	March	Winter	<28	3	0.07 ± 0.09
	Nettle	Female	RS1	Hip/thigh	02/10/2017	February	Winter	<61	7	0.10 ± 0.11
			LS1	Hip/thigh	04/12/2017	April	Spring	<42	6	0.14 ± 0.12
			RS2	Hip/thigh	06/08/2017	June	Spring	<111	6	0.07 ± 0.07
			LS2	Side	08/02/2017	August	Summer	<56	4	0.10 ± 0.10
			RS3	Hip/thigh	09/27/2017	September	Fall	<71	8	0.07 ± 0.08
			LS3	Base of tail	11/22/2017	November	Fall	In progress ^4^		
			RS4	Close to base of tail	01/22/2018	January	Winter	-- ^4^		
			LS4	--	03/19/2018	March	Winter	-- ^4^		

^1^ Shaves ID’s are abbreviated by side and number (e.g., RS1 = Right Shave 1). ^2^ Season is defined by day length: Winter falls between the winter solstice and spring equinox; spring falls between the spring equinox and summer solstice; and summer falls between the summer solstice and the fall equinox. ^3^ It is highly likely that Poblano chewed on his regrowing fur. This sample was excluded from statistical analyses. ^4^ Nettle incurred a mortal self-inflicted injury in January 2018. ^5^ Daily fur growth rates average measured regrowth across biweekly growth checks for each shave for each individual (see Supplementary Table S4).

**Table 3:** Summary regrowth rates for each shave and each species. Dates are presented as Month/Day/Year. More detailed information is provided in Supplementary Table S4.

Taxon	Shave	Date	Time to Full Regrowth (days)			
			Mean	±1σ	Min	Max
*Cheirogaleus*	Right 1	02/10/2017	103.25	25.75	75	131
	Left 1	04/12/2017	66.75	20.84	42	84
	Right 2	06/08/2017	51.00	13.93	40	69
	Left 2	08/02/2017	234.75	130.02	42	327
	Right 3	09/27/2017	247.00	17.49	229	271
	Left 3	11/22/2017	183.75	23.96	159	215
	Right 4	01/22/2018	122.75	13.50	112	140
	Left 4	03/19/2018	74.00	17.49	56	98
	**Total**		**135.40**	**85.10**	**40**	**327**
*Microcebus*	Right 1	02/10/2017	96.25	33.54	61	131
	Left 1	04/12/2017	80.25	57.48	28	154
	Right 2	06/08/2017	86.50	21.00	69	111
	Left 2	08/02/2017	35.00	18.07	14	56
	Right 3	09/27/2017	86.33	67.32	28	160
	Left 3	11/22/2017	159.33	63.72	104	229
	Right 4	01/22/2018	75.00	28.58	43	98
	Left 4	03/19/2018	91.00	89.10	28	154
	**Total**		**86.50**	**51.80**	**14**	**229**

## Supplementary Materials

The following are available online at https://www.mdpi.com/2076-2615/10/8/1288/s1, Table S1: Monthly body weight (g) measurements for study subjects, Table S2: Observations suggesting reduced metabolism and torpor for dwarf lemurs included in this study, Table S3: Photoperiod schedule for DLC nocturnal lemurs in 2017, Table S4: Summary of observed fur regrowth for each shave and individual, Table S5: Results from linear mixed models examining the effects of species, sex, and season or month on cheirogaleid fur regrowth, Table S6: Results from linear mixed models examining the effects of shave location, or the combined effect of location and species, on cheirogaleid fur regrowth.
